# Editorial: Novel biomarkers of cranial nerve-associated diseases identified by multi-omics analysis

**DOI:** 10.3389/fneur.2023.1295470

**Published:** 2023-11-21

**Authors:** Xuefei Song, Weitian Tong, Jian He

**Affiliations:** ^1^Department of Ophthalmolgy, Shanghai Ninth People's Hospital, Shanghai, China; ^2^Shanghai Key Laboratory of Orbital Diseases and Ocular Oncology, Shanghai, China; ^3^Georgia Southern University, Statesboro, GA, United States; ^4^State Key Laboratory of Systems Medicine for Cancer, Center for Single-Cell Omics, School of Public Health, Shanghai Jiao Tong University School of Medicine, Shanghai, China

**Keywords:** biomakers, cranial nerve, multi-omics, optic nerve, radiological characterics

The landscape of cranial nerve-related disorders is extensive, and advancements in technology have enabled researchers to gather metrics from various dimensions, providing a comprehensive understanding of these conditions. With the growing interest in multi-omics research, new perspectives have emerged, leading to the discovery of innovative biomarkers and enhancing our knowledge in disease diagnosis and treatment. This Research Topic serves as a dedicated platform for sharing cutting-edge multi-omics analyses and emerging biomarker research specific to cranial nerve-related disorders. The Research Topic includes seven original research contributions.

Cranial nerve-related disorders are most evident in the study of prevalent neurology conditions. Qi et al. conducted a rigorous examination of biomarkers relevant to patients with ischemic stroke. Their findings revealed that individuals with ischemic cerebral infarction had significantly lower plasma levels of immunoglobulin G neutralizing antibodies (IgG NAbs) against all six peptide antigens examined. Specifically, IgG assays concerning the combinations of IL6-IL8-LOX1a-LOX1b and VEGFR1a-VEGFR1b-LOX1a-LOX1b were identified as efficacious biomarkers for categorizing a distinct subgroup of ischemic stroke patients, thereby facilitating the advancement of precision treatment for the condition. In Wu et al.'s study, a correlation was observed between serum 25-hydroxyvitamin D (25(OH)D) levels and the severity of short-term residual dizziness (RD) in patients diagnosed with benign paroxysmal positional vertigo following successful repositioning maneuvers. Particularly, decreased 25(OH)D levels were associated with an increased likelihood of experiencing moderate to severe RD 1 week after successful repositioning, especially in early-onset female patients. By employing bioinformatics research methodologies, Liu H. et al. predicted tumor outcomes and immune characteristics based on cytokine profiles, providing an avenue for exploring the immunological microenvironment in neural system tumors. Specifically, they developed a Transforming Growth Factor-β (TGF-β) risk model, which exhibited independent prognostic predictive score for glioblastoma. The TGF-β risk score exhibited a robust association with clinical outcomes and tumor microenvironment immune features, making it a valuable metric for evaluating clinical responses to chemotherapy in glioblastoma patients.

The study of cranial nerve-associated disorders also places a significant focus on diseases of the visual system, as evidenced by the three articles included in this Research Topic. Liu X. et al. offer innovative perspectives by examining and evaluating patients through the lens of adjuvant replacement and optic disc blood flow. Their work opens up new avenues for diagnostic and treatment decisions for patients with traumatic optic neuropathy (TON). They identified that the times of dressing changes after the operation is a critical prognostic factor in patients undergoing endoscopic transnasal optic canal decompression (ETOCD). Furthermore, by utilizing optical coherence tomography angiography, they discovered that the microvessel density in the center of the optic disc and superior macula also significantly influences prognostic outcomes. Zhang et al. focus on the impact of specific surgical techniques on treatment efficacy, and their utilization of machine-learning methods is impressive. They provided quantitative evidence that vertical implantation of implantable collamer lenses (ICL) can effectively reduce the achieved vault height, especially when large-size ICLs are implanted, as compared to traditional horizontal implantation methods. For predicting vault height, machine-learning-based models outperform traditional multivariate regression models. Beyond the ophthalmological perspective, Jiang et al. investigate eye diseases from the perspectives of neuroscience and medical imaging. Their research offers new directions for elucidating pathogenic mechanisms by studying regional spontaneous neuronal activity and functional connectivity. Utilizing resting-state functional magnetic resonance imaging (rs-fMRI), they assessed alterations in spontaneous neuronal activity and functional connectivity patterns in Thyroid Eye Disease (TED) patients. Their findings indicate that brain regions associated with motor control and coordination, specifically located in the cerebellum and basal ganglia, exhibit significant compensatory mechanisms in active TED patients.

Additionally, this journal encompasses research findings in otolaryngology. Du et al. address the challenge of differentiating between inverted papilloma (IP) and nasal polyps (NP) through MRI imaging and clinical examinations. They developed a radiomic model based on preoperative multi-modal MRI parameters that demonstrates an efficacious distinction between IP and NP infiltrating the olfactory nerve as a valuable augmentation to standard clinical procedures.

As the editors for this Research Topic, we would like to express our profound gratitude to all contributing authors for their generous submissions. We also extend our appreciation to the experts who have participated in the peer-review process for this Research Topic. Their Collective efforts have played a crucial role in facilitating the successful publication of these high-caliber articles. Furthermore, we are indebted to the staff at *Frontiers in Neurology* for their substantial assistance and guidance throughout every stage of preparation up until now. Our aspiration is that this Research Topic will provide novel insights to researchers in the field of Cranial Nerve-associated Diseases, while simultaneously fostering interdisciplinary research integration and advancement.

## Author contributions

XS: Writing — original draft. WT: Writing — review & editing. JH: Writing — review & editing, Writing — original draft.

